# Process optimization in micro electrical discharge machining of carbon kevlar hybrid composite using TOPSIS

**DOI:** 10.1038/s41598-025-23206-5

**Published:** 2025-11-07

**Authors:** Hrishikesh Dutta, Kishore Debnath, Sargunan Karunanithi, Ashwin Anantharamakrishnan Vimala, Dhinakaran Veeman, Mridusmita Roy Choudhury

**Affiliations:** 1 Centre for Additive Manufacturing, Chennai Institute of Technology, Chennai, 600069 Tamil Nadu India; 2 Department of Mechanical Engineering, National Institute of Technology Meghalaya, Sohra, Meghalaya, 793108 India; 3 Department of Mechanical Engineering, Chennai Institute of Technology, Chennai, 600069 Tamil Nadu India; 4 Department of Mechanical and Industrial Engineering, Manipal Institute of Technology Bengaluru, Manipal Academy of Higher Education, Manipal, 576104 Karnataka India

**Keywords:** Carbon-Kevlar hybrid composite, Micro-electrical discharge machining, Micro-hole, ANOVA, TOPSIS, Morphology., Mechanical engineering, Aerospace engineering

## Abstract

This work is an experimental investigation and multi-response optimization of micro-electrical discharge machining (µEDM) of carbon-Kevlar hybrid composite (CKHC). Based on the chosen input factors namely voltage (V), EDM feed (EF) and tool speed (TS), the experimental runs were fixed as per Taguchi’s L9 orthogonal array. Single and multi-response optimization were carried out using Taguchi and technique for order of preference by similarity to ideal solution (TOPSIS). The responses selected for the study were machining time (MT) and degree of circularity (DOC). The Taguchi analysis identified the optimal input parameter values for minimal MT as a V of 180 volts, EF of 2 μm/sec, and TS of 1000 rpm. The corresponding values for the maximum DOC were 120 volts, 2 μm/sec, and 1000 rpm. Analysis of variance (ANOVA) revealed that voltage significantly affects the variation in MT. However, the DOC is mostly affected by the change in TS. The optimal values of V, EF, and TS obtained from TOPSIS were 150 volts, 4 μm/sec, and 1000 rpm. An improvement of 0.757881 in the preferred solution for optimal settings was confirmed from the analysis. Images from scanning electron microscopy (SEM) confirmed the overcut of the machined micro-holes.

## Introduction

The contemporary industrial landscape needs high-performance materials to explore many potential uses of fiber-reinforced polymer composites (FRPC), and optimizing the performance and weight of the composites can assist in meeting this requirement. The FRPCs are manufactured with the addition of synthetic or natural fibers to a polymer matrix. In the category of synthetic fiber-reinforced composite, carbon fiber-reinforced composites have favorable stiffness, good strength, and low weight, but low impact-resisting properties, low level of energy absorption, and inferior resistance to delamination^1,2^. However, synthetic aramid fibers show a 2.76 GPa tensile strength in addition to significantly improved energy absorption and impact resistance^3,4^. The aerospace, automotive, military, and aircraft industries make extensive use of carbon fiber-reinforced polymer (CFRP) and its hybrid composites as lightweight structural materials because of its many advantages, including better specific strength, strong fatigue resistance, and low weight^5,6^. Similar to this, aramid fiber-reinforced polymer (AFRP) is extensively utilized in the aircraft and space, military, and automotive sectors because of its noticeably improved stiffness, higher specific strength, fatigue resistance, impact resistance, and corrosion resistance^7,8^. Kevlar, the most prevalent para-aramid fiber, exhibits an impact resistance five times better than carbon fiber. Hybrid FRPCs containing more than one type of fiber may be more beneficial than conventional monolithic FRPCs due to the effect of hybridization^9^.

In order to compensate for the disadvantages associated with carbon and Kevlar fibers, researches have been done to incorporate these two fibers in a single laminated composite and study the performance of the resulting carbon-Kevlar hybrid composite (CKHC) in terms of various properties^1,9^. Drilling is the most commonly used technique for machining FRPCs. But there are a few prevailing hurdles associated with the drilling of CFRP and AFRP, namely, delamination, uncut fibres, and burr formation^10–12^. By using non-traditional machining methods like electric discharge machining (EDM) and its variant micro-EDM (µEDM), these problems can be resolved. In recent years, the EDM process has been extensively utilized by researchers for machining various metals and their alloys. Dewan and Kundu^13^ conducted powder-mixed EDM (PMEDM) on Nimonic C-263 to evaluate machining performance in terms of material removal rate (MRR), average surface roughness (R_a_), tool wear rate (TWR), and EDM ratio (ER). With the increase in peak current (I_pc_), pulse on time (T_on_), and pulse off time (T_off_), MRR increased. The maximum value of MRR (2.18378 mm^3^/min) was obtained at 12 A peak current (I_pc_) and 12 g/liter powder concentration (C). The minimum TWR (0.002455 mm³/min) was observed with a powder concentration of 12 g/liter and a T_off_ of 7 µsec. The minimum R_a_ (1.182 μm) was recorded at 9 g/lit C and 7 µsec T_off_. The images obtained from scanning electron microscope (SEM) demonstrated increased crater sizes and debris globules at higher I_pc_ levels. Debnath et al.^14^ studied the impact of voltage, T_on_, capacitance, and tool speed on MRR and machining time (MT) during µEDM of Monel K500 alloy. The value of MRR at 160 volts was 7.73 × 10^−5^ g/min and it increased by 50.06% (11.60 × 10^−5^ g/min) when the voltage was changed to 220 volts for the same values of tool speed (200 rpm), capacitance (100 pF), T_on_ (5 µsec). The optimal values of MRR and MT were 35.68 × 10^−5^ g/min and 16.19 min at a voltage of 220 volts, capacitance of 10,000 pF, tool speed of 400 rpm, and T_on_ of 20 µs.

The optimization of processing parameters during EDM is crucial for achieving a standard set of machining conditions. Kang et al.^15^ used grey relational analysis (GRA) while machining titanium alloy via EDM. The responses selected for the study were MRR, relative electrode wear ratio (REWR), surface roughness (R_a_), and width of cut. The optimal input factors were revealed to be a current of 2 A current, gas pressure of 0.2 MPa, duty cycle of 40% duty, and atomization rate of 20 ml/min. Dewan and Kundu^16^ utilized technique for order preference by similarity to ideal solution (TOPSIS) to optimize process parameters during PMEDM of Nimonic C-263. The optimal parametric settings were identified as - T_on_ = 4 µsec, I_pc_ = 10 A, T_off_ = 5 µsec, flushing pressure = 1 kg/cm², and C = 9 g/liter. SEM micrographs demonstrated that an improved surface finish could be attained by elevating the powder concentration in the dielectric at reduced peak current levels. Chandra et al.^17^ performed Taguchi optimization while machining Aluminum metal matrix composite using EDM. The input parameters were optimized to maximize MRR and minimize TWR using a Taguchi L9 orthogonal array. The machining findings demonstrated that MRR increased with an increase in the pulse current for all the specimens. multi-criteria decision making (MCDM) techniques were utilized by Das et al.^18^ for optimization of µEDM process variables. The best performance of the MCDM - polynomial regression - teaching-learning-based optimization approaches was achieved after assigning 50% weight to MRR. Tripathy and Tripathy^19^ utilized GRA and TOPSIS while machining H aa steel using particle-mixed EDM process. The impact of I_pc_, powder concentration (PC), gap voltage (V), T_on_, and duty cycle (DC) on MRR, TWR, surface roughness (SR), and electrode wear ratio was investigated. The confirmatory test indicates that the enhancement of preferred values in the experimental and initial settings utilizing GRA and TOPSIS is 0.1843 and 0.14308, respectively. The pairs of optimal values of I_pc_, PC, V, T_on_, and DC obtained from GRA and TIOPSIS were (3 A, 6 A), (6 g/L, 6 g/L), (30 V, 50 V), and (100 µs, 100 µs).

In addition to metals and alloys, EDM has been experimented with many non-conductive or partially conductive materials as well. CFRP is one such material that numerous researchers have employed to examine machining performance through the EDM technique. Bajoria et al.^20^ used wire EDM (WEDM) for cutting holes in CFRP. A full factorial experimental design was utilized, incorporating varied ignition current (3 A and 5 A) and T_off_ (4 µsec and 8 µsec). The results indicated that the multidirectional lay-up attained a MRR of 2.85 mm³/min, markedly surpassing the unidirectional lay-up’s MRR of 0.95 mm³/min, signifying a 300% enhancement at 5 A and 4 µsec. The augmentation in discharge energy resulted in surface degradation, including delamination, frayed fibers, and uneven circularity, particularly noticeable in the unidirectional lay-up. Pattanayak et al.^21^ studied the performance of CFRP during the fabrication of holes using EDM process. The statistical study indicated that the pulse-on time exerts a more significant influence than flushing pressure, current, and pulse-off time. The hole corresponding to the optimized machining conditions exhibits high circularity, low electrode wear, and a low angle of taper. Singh et al.^22^ adopted Taguchi’s L9 orthogonal array for machining CFRP using electro chemical discharge machining (ECDM) technique. Effect of voltage, electrolyte concentration, and interelectrode gap on MRR and overcut was investigated. The results of TOPSIS revelated improvements in MRR from 2.232 mg/min to 2.1267 mg/min and overcut from 150 μm to 48 μm. An Entropy weightage method combined with TOPSIS was utilized by Yadav et al.^23^ for simultaneous optimization of overcut and hole taper in ECDM of CFRP. The optimal values of voltage, tool speed, DC, and tool travel rate for the optimum combination of overcut and taper were 50 V, 360 rpm, 60%, and 0.5 mm/min^−1^, respectively.

Although carbon-Kevlar hybrid composites are increasingly utilized in aerospace, defense, and medicinal fields, the micromachining of these materials via µEDM is still completely unexamined due to their intricate anisotropic and thermally resilient structure. The existing literature primarily concentrates on traditional machining or EDM of singular carbon or aramid fibers, with scant attention given to hybrid laminates. Furthermore, the majority of research focus on single-response optimization, neglecting the interrelated characteristics of performance metrics such as material removal rate (MRR), tool wear rate (TWR), and surface integrity. This study addresses this gap by utilizing a multi-response optimization method through TOPSIS, facilitating a comprehensive assessment of process parameters. The incorporation of TOPSIS facilitates a robust, rank-oriented decision-making framework, resolving competing machining objectives and providing practical insights into the efficient micro-EDM of CKHC, a field that is devoid of systematic, data-driven optimization methodologies.

This work is a comprehensive study on the parametric optimization of µEDM of CKHC. After using Taguchi’s L9 orthogonal array as the experiment’s design, the effects of several machining parameters, including voltage, tool speed, and EDM feed rate, on machining time (MT) and degree of circularity (DOC) were evaluated. The results were analyzed using the statistical analysis tool ‘*analysis of variance* (ANOVA)’. In addition to Taguchi optimization, TOPSIS was used for the simultaneous optimization of MT and DOC. The results of this study will serve as the foundation for future research endeavors in the area of non-conventional machining of polymer composites containing synthetic fibers which are electrically non-conductive.

### Novelty and significance

In contrast to CFRP or other composites containing single type of fiber, CKHC poses a distinct difficulty owing to the combination of carbon and Kevlar fibers, which exhibit significantly different thermal and electrical attributes. These divergent properties affect the machining of CKHC via µEDM in terms of ease of spark generation, quality of micro-feature, and the heat-affected zone in fundamentally distinct manners relative to CFRP and other electrically-conductive materials. This study is a novel attempt to provide insights into the optimization of µEDM process parameters through single and multi-response optimization techniques including Taguchi and TOPSIS, which has not been done before in context of µEDM of CKHC. The work presents a processability standard for CKHC across several micro-EDM parameters, emphasizing ideal parameter ranges where machining efficiency and surface quality align. This is especially beneficial for microfabrication sectors investigating lightweight multifunctional composites. A notable significance of this study is the inclusion of DOC as a critical response variable, enabling quantitative assessment of the reliability of micro-feature fidelity and dimensional accuracy, which has seldom been addressed in the micromachining of hybrid composites.

## Materials and methods

### Materials

The supplier of the 3 K plain weave carbon fabrics utilized in the study was FIBER SOURCE, located in Chennai, India. The fabric had a thickness of 0.19 ± 0.005 mm and a thread count of 5.0 ends/cm for both WEFT and WARP. The carbon fiber has a modulus of 238 GPa and a tensile strength of 3950 MPa, as mentioned in the maker’s (TOHO TENAX) specification details. The same supplier also provided the bi-directional Kevlar fabrics, which are para-aramid fibers. The Kevlar fabric had a simple weave and a thickness of 0.22 mm. According to the material’s technical data sheet, the Kevlar fiber’s breaking strength and breaking tenacity were 226 N and 2.92 GPa, respectively. The hybrid composite laminates were fabricated using an epoxy resin system that included HY951 and LY 556.

### Fabrication of CKHC

**Fig. 1:**
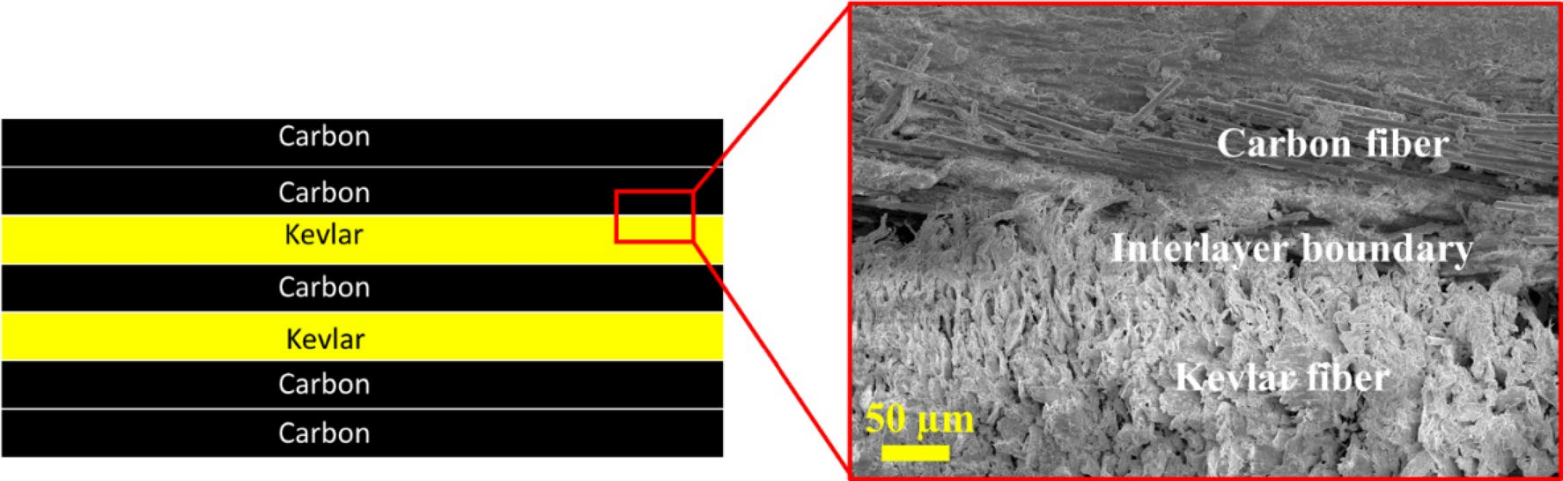
Schematic representation of the CKHC laminate along with the microscopic view of the interlayer region.

The hybrid interlayer laminates were created using the vacuum assisted resin transfer moulding (VARTM) technique. Initially, the carbon and Kevlar fabrics were cut into rectangular shape of 20 cm × 15 cm. then five layers of carbon and two layers of Kevlar were stacked as per the arrangement shown in Fig. 1. this stack of fabrics is put inside the vacuum bagging set up and resin is infused into the stack using VARTM technique. The composite laminate left to cure overnight in room temperature. The microscopic view of the interlayer region is also shown in the figure. The layers of carbon and Kevlar fibers are clearly visible on both sides of the interlayer boundary.

### Experimentation

**Fig. 2:**
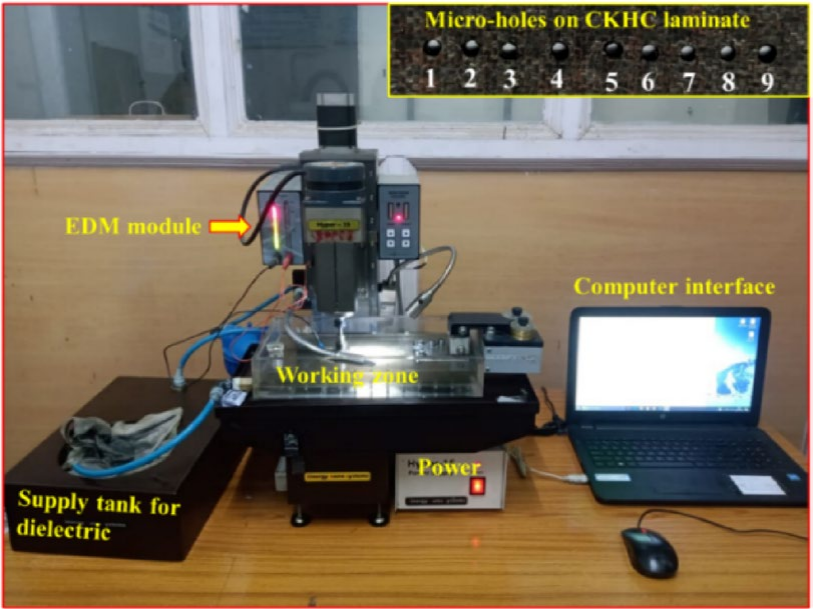
Hyper-15 micro-machining set up with the carbon Kevlar hybrid composite (CKHC) laminate showing micro-holes.

The micro-hole fabrication on CKHC laminate was conducted utilizing the HYPER 15 micro-machining equipment manufactured by Sinergy Nano Systems (Fig. 2). The work table of the machine can travel up to a maximum span of 130 mm, 75 mm, and 80 mm in the X, Y, and Z directions, respectively. A copper tool of 800 μm diameter was used for the machining. Based on earlier studies^14,24^ relevant to the present topic, three input parameters, viz., voltage, tool speed, and EDM feed, were considered for the study. The experimental runs were generated using Taguchi’s L9 orthogonal array based on the selected input factors. The selection of Taguchi L9 orthogonal array was motivated by the need for a fast optimization process with minimal experimental runs involving reduced cost. However, this design restricts the analysis of interaction effects among process factors. Future research should employ higher-order factorial or response surface approaches to more thoroughly elucidate parameter interactions and augment process comprehension for CKHC micromachining. Table 1 displays the levels and values of the parameters. Regression analysis using an ANOVA with a 95% confidence level was used to determine the input parameter’s significant level. Minitab 17 was also used for the Taguchi method-based optimization procedure.

**Table 1:** Machining factors and their values at different levels.

Factors	Unit	Level 1	Level 2	Level 3
		Values		
Voltage (V)	volts	120	150	180
Tool speed (TS)	rpm	400	700	1000
EDM feed (EF)	µm/sec	2	4	6

### Measurement of machining time and degree of circularity (DOC)

**Fig. 3:**
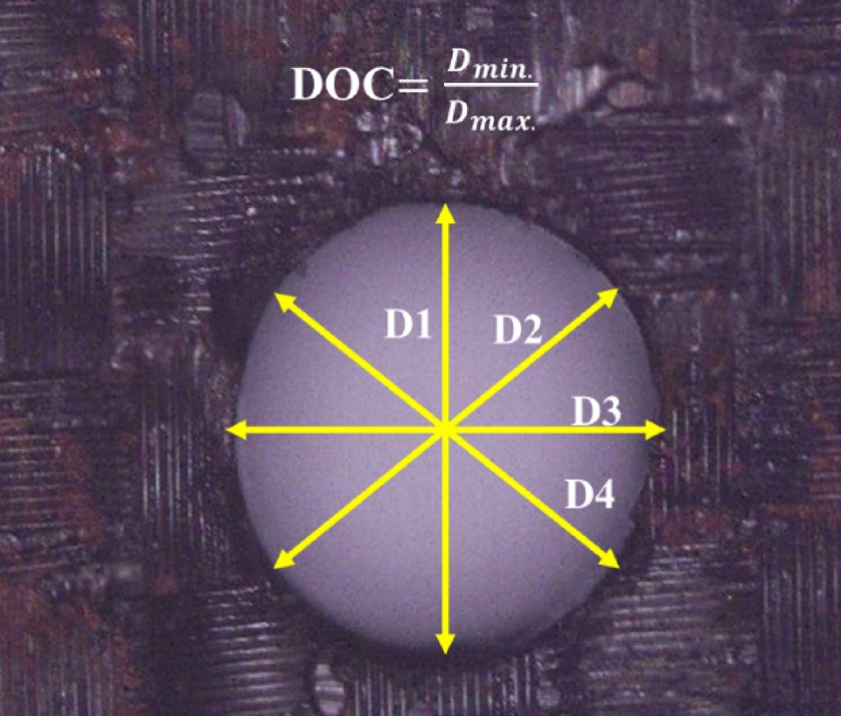
Process of measuring degree of circularity (DOC) as the ratio between minimum diameter (D_min_) and maximum diameter (D_max_).

The machining time was measured using a stopwatch. The process of measuring DOC is shown schematically in Fig. 3. Initially the micro-hole is focused properly under an optical microscope. Then, the approximate location of the Centre of the hole is fixed. Four straight lines are drawn passing through the centre and touching the opposite ends of along the circumference of the hole. Due to the presence of overcut regions around the hole these diameters will have different length. The DOC is calculated by taking the ratio of the minimum diameter (D_min_) to the maximum diameter (D_max_) found from the measurement. Although this method of measuring DOC is straightforward and practical, it is apparent that there will be some uncertainty and operator-induced variability in the result. Therefore, it is recommended to evaluate the method’s repeatability through several measurements under identical conditions and to employ modern image processing technologies for enhanced consistency and accuracy.

### Technique for order of preference by similarity to ideal solution

TOPSIS is a multi-criteria decision-making that operates on the notion that the chosen alternatives should exhibit the minimal distance from the positive ideal solution while maximizing the distance from the negative ideal solution^19^. The TOPSIS approach is recognized for its efficacy and computational simplicity in evaluating and selecting the appropriate parameters from a group of possibilities. This method employs the criteria measurements along with their relative significance to determine the final ranking of the options. The method relies on the notion that the selected criteria should exhibit the minimal distance from the positive ideal solution and the maximal distance from the negative ideal solution, with the optimal option being the one that demonstrates the highest relative proximity to the ideal answer. The technique for executing TOPSIS can be delineated as follows:

Step 1: The first step of TOPSIS is the representation of the *m×n* decision matrix (d_m_), where m is the number of alternatives and n is the number of attributes.

$$\:{d_m} = \:\left[ {\begin{array}{*{20}{c}} {{y_{11}}}&{\:{y_{12}}}&{\:{y_{13}}}&{\: \cdots \:}&{\: \cdots \:}&{\:{y_{1n}}} \\ {\:{y_{21}}}&{\:{y_{22}}}&{\:{y_{23}}}&{\: \cdots \:}&{\: \cdots \:}&{\:{y_{2n}}} \\ {\:{y_{31}}}&{\:{y_{32}}}&{\:{y_{33}}}&{\: \cdots \:}&{\: \cdots \:}&{\:{y_{3n}}} \\ \vdots &{\: \vdots }&{\: \vdots }&{\: \ddots \:}&{\: \ddots \:}&{\: \vdots } \\ {\: \vdots }&{\: \vdots }&{\: \vdots }&{\: \ddots \:}&{\: \ddots \:}&{\: \vdots } \\ {\:{y_{m1}}}&{\:{y_{m2}}}&{\:{y_{m3}}}&{\: \cdots \:}&{\: \cdots \:}&{\:{y_{mn}}} \end{array}} \right]\;$$

Step 2: Normalization of the observed data using following equation:

1 $$\:{Z}_{ij}=\frac{{y}_{ij}}{{\left({\sum\:}_{i=1}^{m}{y}_{ij}^{2}\right)}^{\frac{1}{2}}}$$

Step 3: Finding the weightage normalized values of the data:

2 $$\:B\:\left({b}_{ij}\right)={w}_{j}{z}_{ij}$$

Step 4: The ideal best (B^+^) and negative ideal (B^−^)values of the responses are selected from the dataset of weightage normalized value (B).

Step 5: The separation measures of each alternative from the ideal and the negative ideal solution are calculated using equations [3] and [4].

3 $$\:{S}_{i}^{+}={\left\{\sum\:_{j=1}^{n}\left({b}_{ij}-{b}_{j}^{+}\right)\right\}}^{\frac{1}{2}}$$

4 $$\:{S}_{i}^{-}={\left\{\sum\:_{j=1}^{n}\left({b}_{ij}-{b}_{j}^{-}\right)\right\}}^{\frac{1}{2}}$$

Step 6: The closeness index (C_i_)denoting the relative closeness of a specific alternative to the ideal value is calculated using following relation:

5 $$\:{C}_{i}=\frac{{S}_{i}^{-}}{{S}_{i}^{+}+{S}_{i}^{-}}$$

## Results and discussion

### Taguchi and analysis of variance (ANOVA)

Table 2 presents the findings from the experiments conducted using Taguchi’s L9 orthogonal array. Since lower MT consistently translates into higher production, the signal-to-noise ratio (S/N ratio) for MT is calculated by taking the “smaller-is-better” criteria. This is due to the fact that smaller machining time results in better productivity by speeding up the manufacturing process. For micro-machining operation better circularity of the holes is always a desirable aspect. Hence, the “larger-is-better” criteria is considered for the calculation of S/N ratio for DOC.

**Fig. 4:**
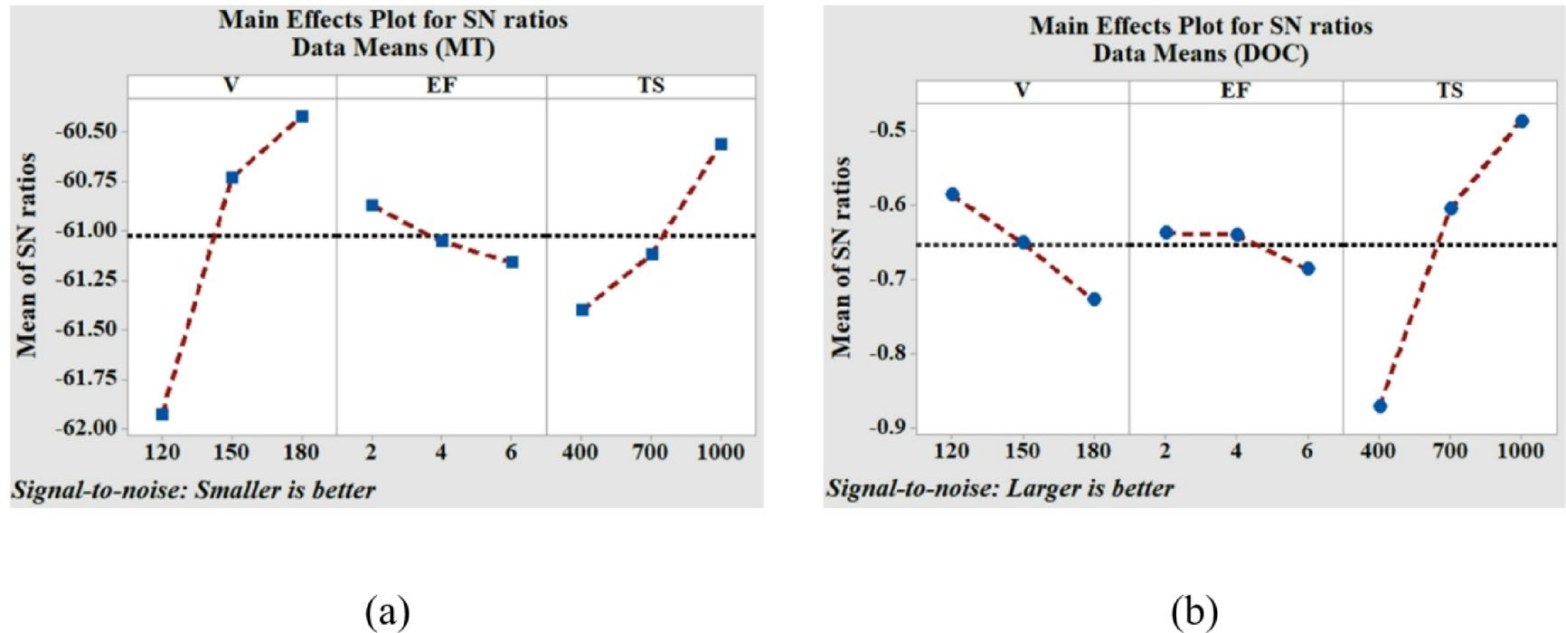
**‘**Mean of S/N ratio’ plot for the input factors for (**a**) machining time (MT), and (**b**) degree of circularity (DOC).

A representation of the intrinsic influence that the input parameters have on the MT and DOC is shown in Fig. 4. The relative inclination of the linear graphs indicates the significance of the machining parameters that are considered for the study. Figure 4(a) clearly demonstrates that the slope of the graph representing the effect of voltage on MT is greater than the slopes of the other two graphs. Hence, it may be inferred that the voltage has a substantial impact on the machining time. While the speed of the tool is relatively important, the pulse duration is the least important parameter. The result demonstrates a positive correlation between the MT and both the voltage and tool speed. This outcome can be explained by the increasing nature of the discharge energy (DE) with voltage during µEDM. The elevated DE generates a greater quantity of heat on the work surface, resulting in faster material removal through the process of melting the composite constituents. The reason for the reduced MT with the increase in tool speed is the enhanced effect of efficient flushing assisted by the movement of the rotating tool. A whirling action is created in the dielectric due to the rotational motion of the tool electrode, which becomes intense at a higher speed. This aids in the effective removal of debris and makes machining faster. It is understood form Fig. 4(b) that TS has the highest impact on the variation of DOC. In µEDM, increasing tool rotational speed may enhance the circularity of the hole by facilitating debris removal and minimizing tool wear. Increased rotational speeds enhance the flushing of the working gap, so avoiding debris from obstructing the spark discharge and resulting in abnormalities. Similar outcomes on the roundness of micro-holes were reported by Kaushik et al.^25^, while studying the µEDM performance of CFRP.

**Fig. 5:**
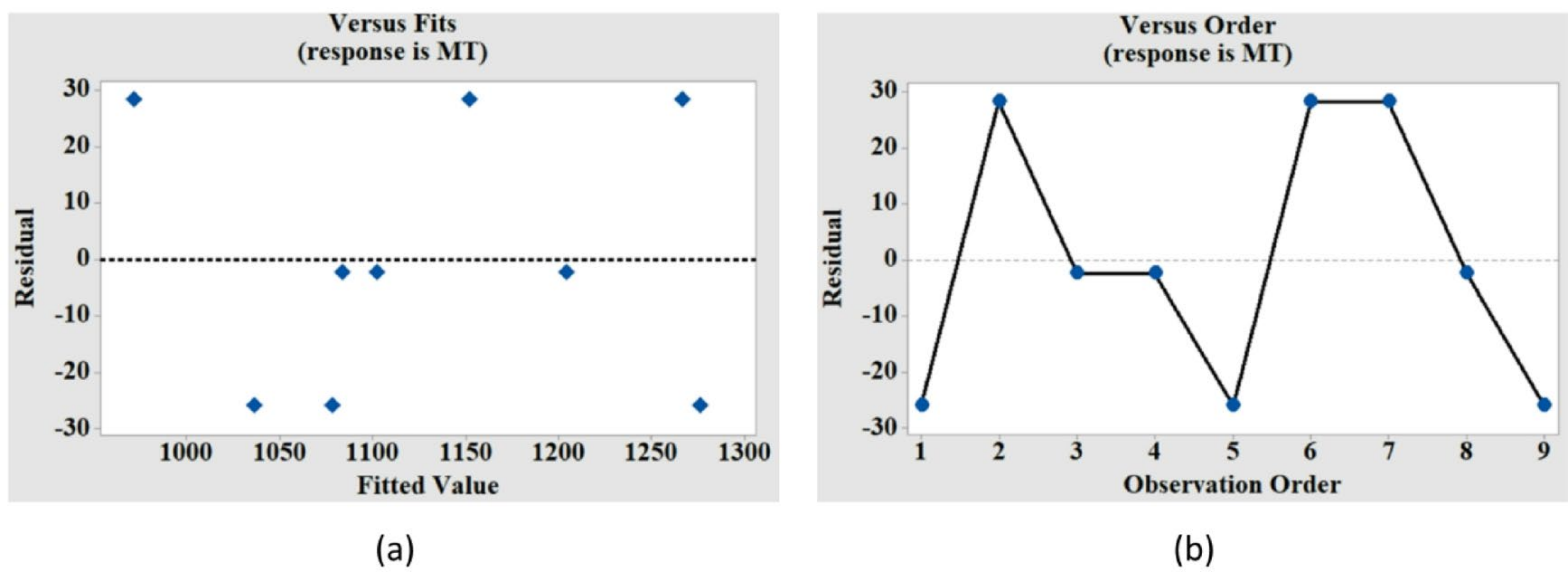
Residual plots for machining time (MT) versus (**a**) fitted values, and (**b**) observation order.

**Fig. 6:**
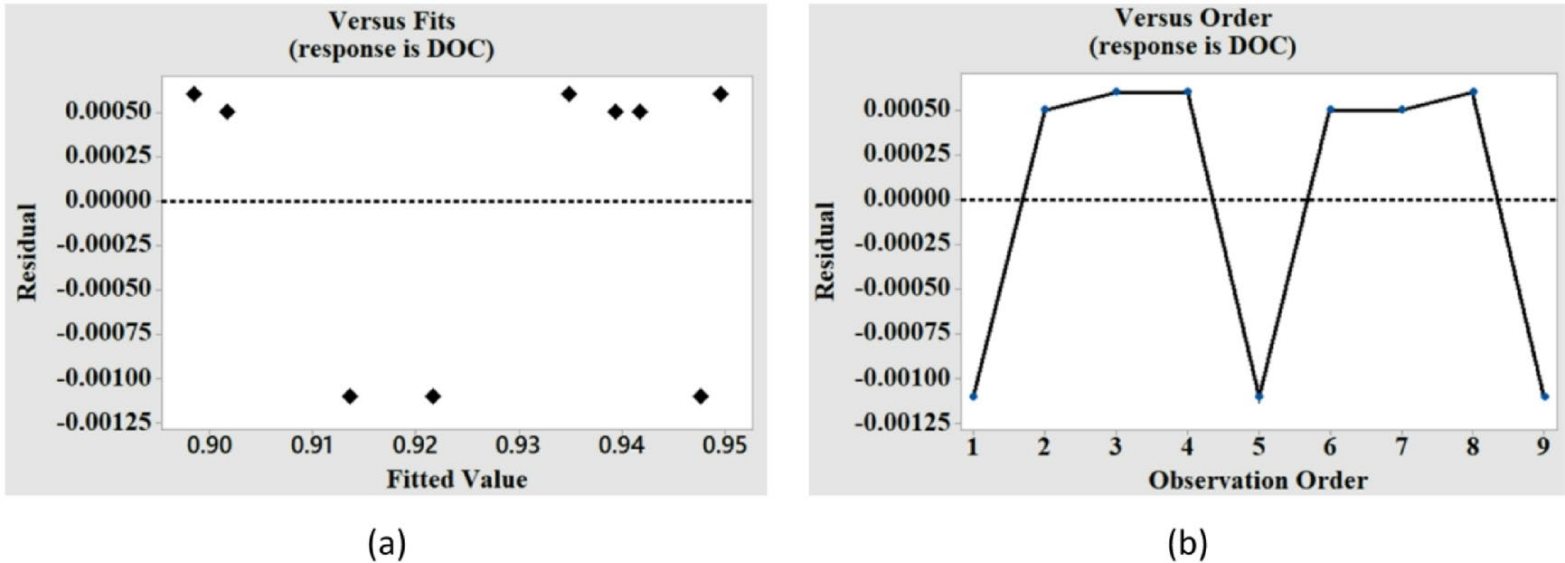
Residual plots for degree of circularity (DOC) versus (**a**) fitted values, and (**b**) observation order.

Table 3 displays the findings of the variance analysis for MT and DOC. The findings of the regression analysis for MT indicate that the impact of voltage and tool speed on the MT variation is significant, which is confirmed by the p-value for these two factors being less than 0.05 (with a 95% confidence interval). The analysis shows that voltage has the largest percentage contribution to the variance of MT. Moreover, the model’s fitness is indicated by the R-sq value close to 1 (0.8626). Voltage and tool speed are proved to be significant for DOC also, as indicated by their p-values (0.016,0.000) in Table 3. As discussed in the earlier section the higher impact of TS on the DOC is reconfirmed by the contribution percentage of TS (82.90%) presented in Table 3. Residual graphs for MT and DOC are presented in Figs. 5 and 6. The residuals versus fits plot are created to validate the assumption of random distribution and homoscedasticity of residuals. The optimal distribution of points must be random on either side of zero, devoid of any discernible patterns^26^. Figure 5(a) illustrates that the data are randomly dispersed around zero, exhibiting no discernible pattern, so corroborating the premise. To validate the hypothesis that the residuals are independent, a residual against order plot was created, as illustrated in Fig. 5(b). The plot demonstrates that the residuals are randomly distributed around the central line, indicating their independence^26^. Similar observations are made from the residual plots for DOC as shown in Fig. 6(a) and Fig. 6(b).

Table 4 displays the response for S/N ratios for MT and DOC. The level corresponding to the highest S/N ratio is observed as the optimal level for that particular factor. Table 5 displays the ideal input factor levels for the minimum MT and maximum DOC. The optimal combinations of input parameters found from Taguchi’s optimization are V_3_EF_1_TS_3_, and V_1_EF_1_TS_3_ for MT and DOC, respectively. The regression models for MT and DOC are represented by Eq. 5 and Eq. 6. The computed and experimental values of MT and DOC at the best possible level of input factors are shown in Table 6. The calculated and experimental machining times differ by as little as 3.96% and 0.93% for MT and DOC, respectively, suggesting that the chosen statistical model performs well in forecasting the chosen responses.

This study uses Taguchi’s L9 orthogonal array with a motive to reduce the number of experimental runs, thereby minimizing the overall duration and cost of experimentation. The primary objective was to assess the main effects of the machining parameters on MT and DOC. However, it is possible that specific interactions among voltage and tool speed may impact the machining performance by a collective effect of energy variation and effective flushing.

**Table 2:** Experimental results with S/N ratios for MT.

Sl. No.	Voltage (volts)	EDM feed (µm/sec)	Tool speed (rpm)	Machining time (sec)	S/*N*_MT_ (db)	Degree of circularity	S/*N*_OC_ (db)
1	120	2	400	1250	−61.9382	0.9245	−0.795343
2	120	4	700	1295	−62.2454	0.9422	−0.517138
3	120	6	1000	1202	−61.5981	0.9502	−0.443699
4	150	2	700	1081	−60.6765	0.9354	−0.580053
5	150	4	1000	1010	−60.0864	0.9465	−0.477588
6	150	6	400	1180	−61.4376	0.9022	−0.893944
7	180	2	1000	1000	−59.0849	0.9399	−0.538367
8	180	4	400	1100	−60.8279	0.899	−0.924806
9	180	6	700	1052	−60.4403	0.9205	−0.719524

**Table 3:** Different results obtained from analysis of variance for machining time (MT) and degree of circularity (DOC).

Source	DF	Adj SS	Adj MS	F-value	*P*-value	%contribution
For MT						
Regression	3	77,626	25,875	10.46	0.014	
V	1	59,004	59,004	23.85	0.005	65.56
EF	1	1768	1768	0.71	0.436	1.96
TS	1	16,854	16,854	6.81	0.048	18.73
Error	5	12,368	2474			13.74
Total	8	89,994				
R-sq = 0.8626; R-sq(adj) = 0.7801						
For DOC						
Regression	3	0.00289	0.00096	35.70	0.001	
V	1	0.00034	0.00034	12.75	0.016	11.18
EF	1	0.00004	0.00004	1.37	0.295	1.32
TS	1	0.00252	0.00252	92.99	0.000	82.90
Error	5	0.00014	0.00003			4.46
Total	8	0.00304				
R-sq = 0.9554; R-sq(adj) = 0.9286						

**Table 4:** S/N ratio’s response for machining time (MT) and degree of circularity (DOC).

	Voltage	EDM Feed	Tool Speed
For MT			
1	−61.93	*−60.87*	−61.40
2	−60.73	−61.05	−61.12
3	*−60.42*	−61.16	*−60.56*
Delta	1.50	0.29	0.84
Rank	1	3	2
For DOC			
1	*−0.5854*	*−0.6379*	−0.8714
2	−0.6505	−0.6398	−0.6056
3	−0.7276	−0.6857	*−0.4866*
Delta	0.1422	0.0478	0.3848
Rank	2	3	1

**Table 5:** Optimal values and corresponding levels of input parameters as per taguchi’s optimization.

Response	Parameters	Unit	Coding	Value	Level
MT	Voltage	volts	V	180	3
	EDM Feed	µm/sec	EF	2	1
	Tool speed	rpm	TS	1000	3
DOC	Voltage	volts	V	120	1
	EDM Feed	µm/sec	EF	2	1
	Tool speed	rpm	TS	1000	3

5 $$MT\;\;\; = \;\;\;1715{\text{ }} - \;3.306\;V{\text{ }} + \;8.6\;EF{\text{ }} - \;0.1767\;TS$$

6 $$DOC\;\; = 0.9227{\text{ }}-\;0.000253\;V{\text{ }} - \;0.00124\;EF{\text{ }} + \;0.000068\;TS\;$$

**Table 6:** Comparison of calculated and experimental machining time (MT) and degree of circularity (DOC) at optimum levels.

Response	Calculated	Experimental	%Error
MT (sec)	960.42	1000	3.96
DOC	0.9578	0.9490	0.93

### Study of dimensional accuracy

**Fig. 7:**
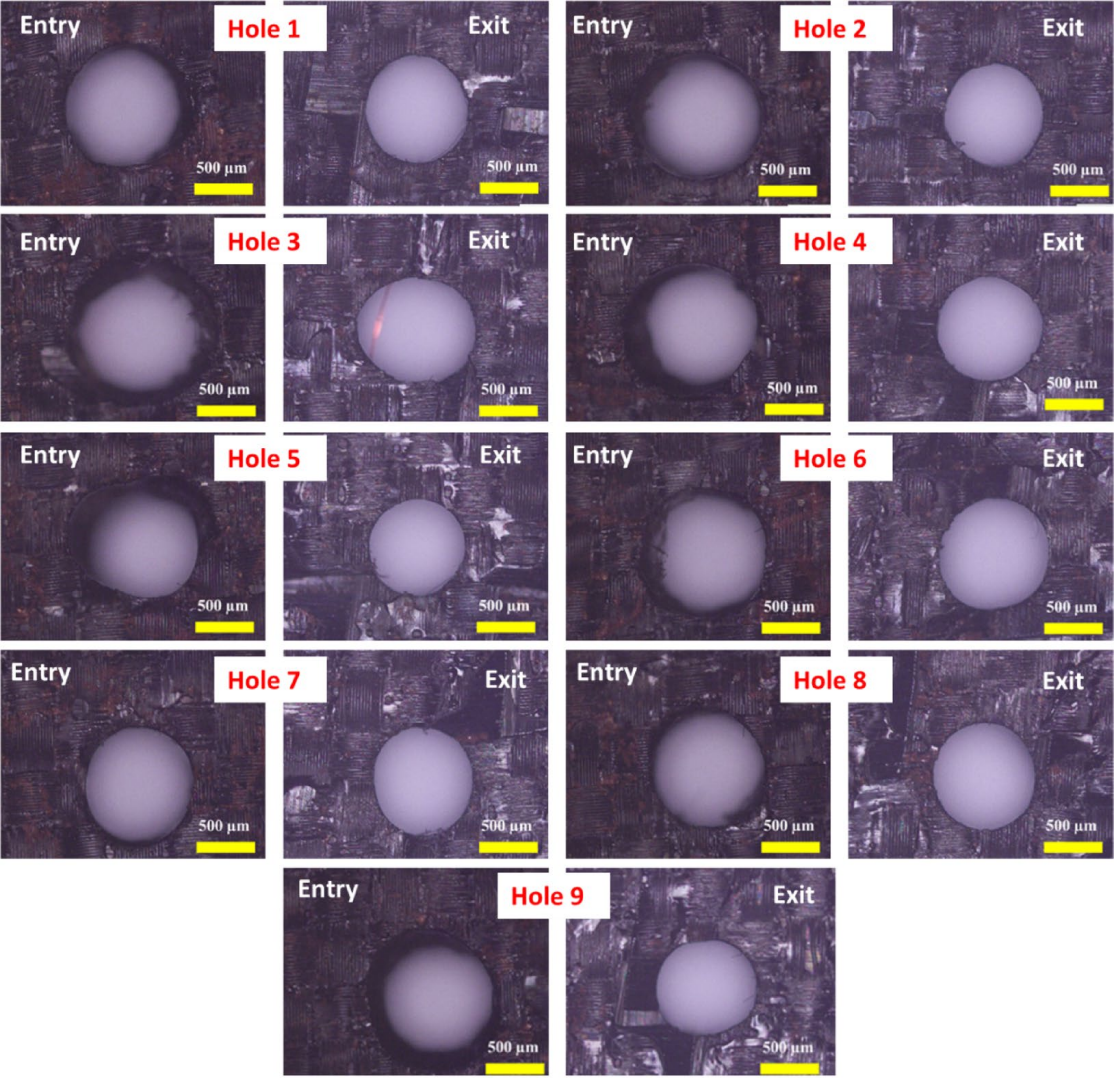
Optical micrographs of fabricated micro-holes at entry and exit.

The dimensional assessment was carried out by analyzing the machined micro-holes under the optical microscope. Figure 7 presents the optical micrographs of the machined micro-holes at entry and exit. The first observation from the micrograph is that the diameter of the hole at the exit is less than that at the entry. This difference in diameter between the entry and exit is evidence of hole tapering, which is generally an unavoidable phenomenon during through-hole fabrication using µEDM^27,28^. Another significant observation is the presence of overcut regions in all the holes around their entries, which clearly distinguishes them from the exit morphology. The unremoved portions around the hole boundary can be seen in some of the holes (hole 2 and hole 6).

**Fig. 8:**
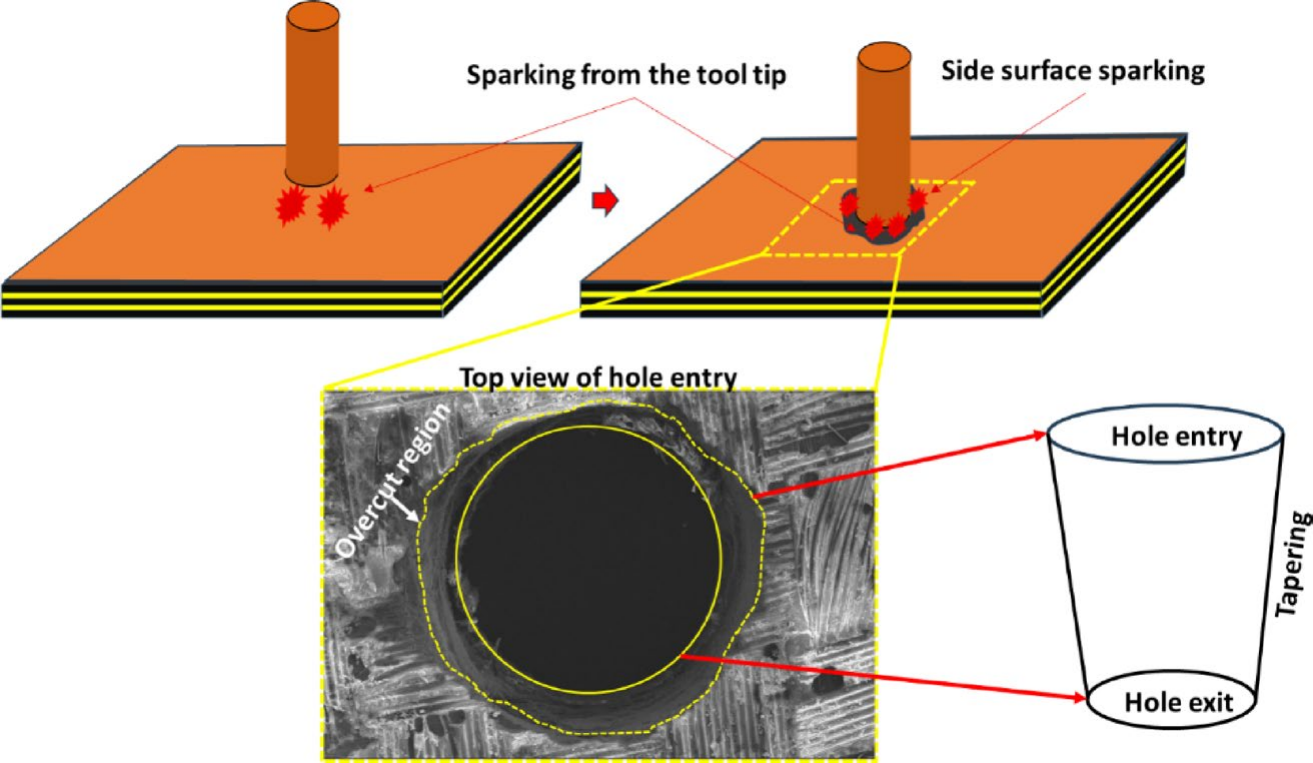
Schematic of overcut due to side-surface-sparking (SS-sparking).

The factor behind the hole-tapering phenomenon is the side-surface-sparking (SS-sparking) during µEDM. The process of SS-sparking and the resulting overcut around the entry of the hole is explained with the help of a schematic and a surface micrograph in Fig. 8. It is evident that sparks are produced between the tool’s tip and the work surface, which is protected from sparking by a conductive layer. As visible in the schematic, once the tool travels a certain distance along the depth of the hole, SS-sparking starts to occur, leading to the removal of access material towards the top side of the hole. This SS-sparking continues till the end of the machining, and finally, the tool crosses the last layer of the laminate and completes the through-hole. The resulting through-hole exhibits a tapering along the depth starting from the entry to the exit of the hole, as shown schematically in Fig. 8. During µEDM the passing of the debris through the narrow side gap between the tool and inner hole surface leads to additional side sparking. This also aids to the tapering of the micro-hole. In an effort to reduce the taper in the micro-hole drilled on CFRP via µEDM, Kumar and Singh^29^ designed a modified solid cylindrical tool with slotted surface. The micro slots made on the tool’s surface were expected aid better flushing of the debris leading to reduced tapering of the hole by minimizing unwanted additional sparking. However, fabrication of such micro-slotted cylindrical tool with the diameter in the range of a micron is a tedious job and needs further extensive investigations. The results of the overcut measurements are presented in Table 7. The overcut in this study has been defined as the difference between the entry and exit diameters of the hole. The minimum and maximum values of overcut are 77.78 μm and 244.44 μm corresponding to hole number 7 and 4, respectively.

**Table 7:** Values of entry diameter, exit diameter, and overcut of the micro-holes.

Hole number	Entry diameter (µm)	Exit diameter (µm)	Overcut (µm)
1	1055.55	888.89	166.67
2	1111.11	844.45	266.66
3	1222.20	1027.78	194.42
4	1138.89	894.45	244.44
5	1061.11	833.33	227.78
6	1100.00	933.33	166.67
7	927.78	850.00	77.78
8	966.67	877.78	88.89
9	1050.00	840.44	209.56

### Process evaluation using TOPSIS

**Fig. 9:**
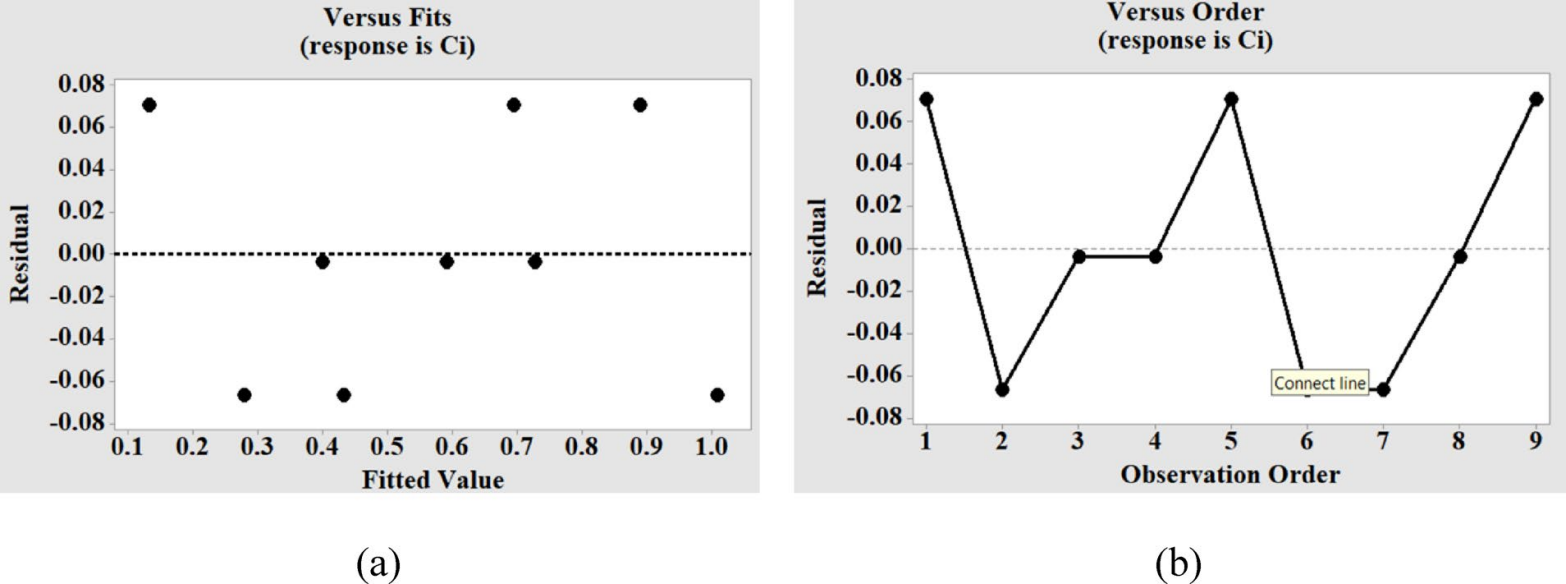
Residual plots for closeness index (C_i_) versus (**a**) fitted values, and (**b**) observation order.

The multi-criteria optimization was conducted with TOPSIS, to find a set of input parameters for the optimum values of MT and OC. The preference value for each experimental combination was calculated using the data from the experimental runs. The normalization of the results is done and their weighted values are calculated considering the assigned weightage for each of the responses. In the current study weightage for DOC and MT were considered as 0.6, and 0.4, respectively. A higher weightage to DOC was given considering its importance in precision micro-machining^22^. This specific allocation method supports the need of geometric accuracy of micro-features in CKHC components. Although the weights for DOC and MT were selected based on application-specific factors, future research should utilize objective weighting methods, such as the entropy method or AHP, to enhance methodological rigor and reduce subjectivity in multi-criteria decision-making. The normalized and weighted normalized values are presented in Table 8. The preference value for each alternative can be computed by evaluating the relative proximity to the ideal solution, defined as the ratio of the negative ideal separation measure to the sum of the negative and positive ideal separation measures. The primary motive of the analysis is to transform the multicriteria optimization problem into a single-objective optimization problem by combining the Taguchi’s design with TOPSIS. The values of separation measures and closeness indices are shown in Table 9. The ranking of the closeness indices is done in order to find out the best possible combination of parameters for optimum MT and DOC. The proximity to the optimum solution values for the optimal performance metric dictates the maximum preference value and highest ranking, thereby serving as the best value and performance measure for the process. The highest value of C_i_ was found to be 0.961043742 for the experimental run 5, as seen in Table 9. Hence, the best combination of input parameters resulted from TOPSIS are V2/EF2/TS3, i.e. voltage of 150 volts, EDM feed of 4 μm/s, and tool speed of 1000 rpm. The assessment of the optimal settings of the input parameters by TOPSIS was followed by the confirmatory analysis to assess the improvement in the ideal solution. As shown in Table 10, an improvement of 0.757881 in the ideal solution could be observed when compared to the initial solution. The ANOVA was carried out for the C_i_ values and the results are presented in Table 11. ANOVA was performed to assess the significance of input parameters in the variation of overall performance characteristic (Ci) of TOPSIS^19^. It aided in identifying statistically important factors that contribute to effective decision-making^22^. The results reveal that voltage has the highest significance on the variation of the closeness indices obtained from TOPSIS. Moreover, a higher value of R-sq (0.8774) confirms the fitness the model for C_i_. The plots for residuals versus fitted values and orders corresponding to C_i_ are shown in Fig. 9(a) and (b). The plots demonstrate random distribution of residuals around the central line confirming equal variance and independence of the residuals.

**Table 8:** Normalized and weighted normalized values of results.

Exp. no.	Normalized values $\:({\varvec{Z}}_{\varvec{i}\varvec{j}})$		Weighted normalized values $\:({\varvec{b}}_{\varvec{i}\varvec{j}})$	
	MT	DOC	MT	DOC
1	0.36729623	0.331682706	0.146918492	0.199009623
2	0.380518894	0.338032932	0.152207558	0.202819759
3	0.353192054	0.34090309	0.141276822	0.204541854
4	0.317637779	0.335593297	0.127055112	0.201355978
5	0.296775354	0.339575642	0.118710141	0.203745385
6	0.346727641	0.323682139	0.138691056	0.194209283
7	0.293836984	0.337207761	0.117534794	0.202324657
8	0.323220682	0.322534075	0.129288273	0.193520445
9	0.309116507	0.330247626	0.123646603	0.198148576

**Table 9:** Separation measures and closeness indices.

Exp. no.	Separation measures		Closeness index (C_i_)	Rank
	$\:{\varvec{S}}_{\varvec{i}}^{+}$	$\:{\varvec{S}}_{\varvec{i}}^{-}$		
1	0.029899779	0.00762331	0.203163186	9
2	0.034715304	0.009299759	0.211285825	8
3	0.023741822	0.01552327	0.39534532	6
4	0.010039087	0.026345215	0.724081915	4
5	0.001419702	0.035023778	0.961043742	1
6	0.023544506	0.013534507	0.365017995	7
7	0.002217343	0.035773639	0.941635008	2
8	0.016112545	0.022919727	0.587199414	5
9	0.008844635	0.028934013	0.765882712	3

**Table 10:** Confirmatory test for TOPSIS.

Factors/Responses	Initial settings	Optimal experimental settings
	V1/EF1/TS1	V2/EF2/TS3
Voltage	120	150
EDM feed	2	4
Tool speed	400	1000
MT	1250	1010
DOC	0.9245	0.9465
Value of preferred solution	0.203163	0.961044
Improvement in C_i_	0.757881	

**Table 11:** Results from analysis of variance (ANOVA) for closeness index (C_i_).

Source	DF	Adj SS	Adj MS	F-Value	*P*-Value
Regression	3	0.60467	0.20156	11.93	0.010
V	1	0.36750	0.36750	21.76	0.006
EF	1	0.01957	0.01957	1.16	0.331
TS	1	0.21761	0.21761	12.88	0.016
Error	5	0.08446	0.01689		
Total	8	0.68913			
R-sq = 0.8774; R-sq(adj) = 0.8039					

### Morphological assessment

**Fig. 10:**
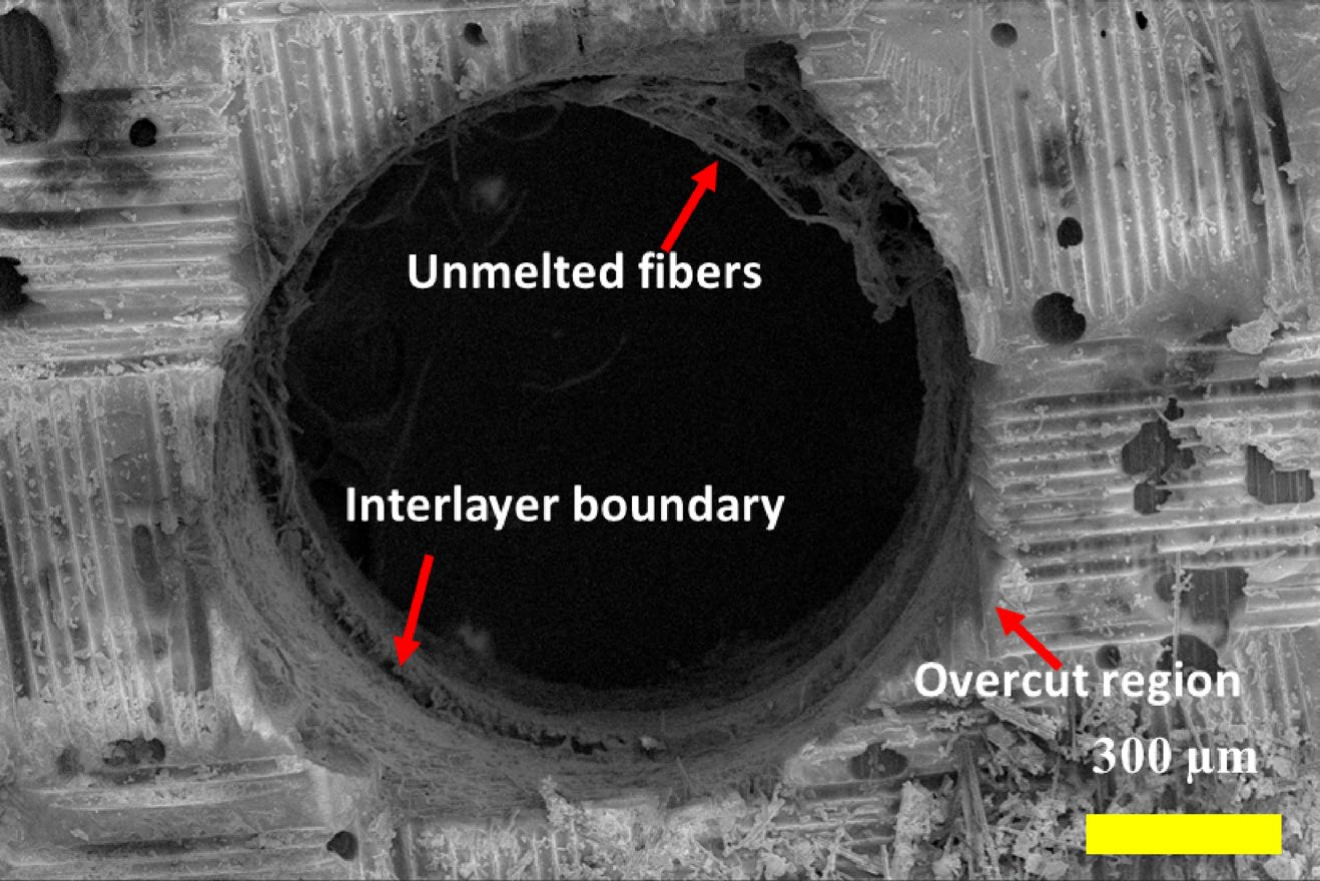
Morphology of the micro-hole showing unmeted fibers and interlayer boundary.

**Fig. 11:**
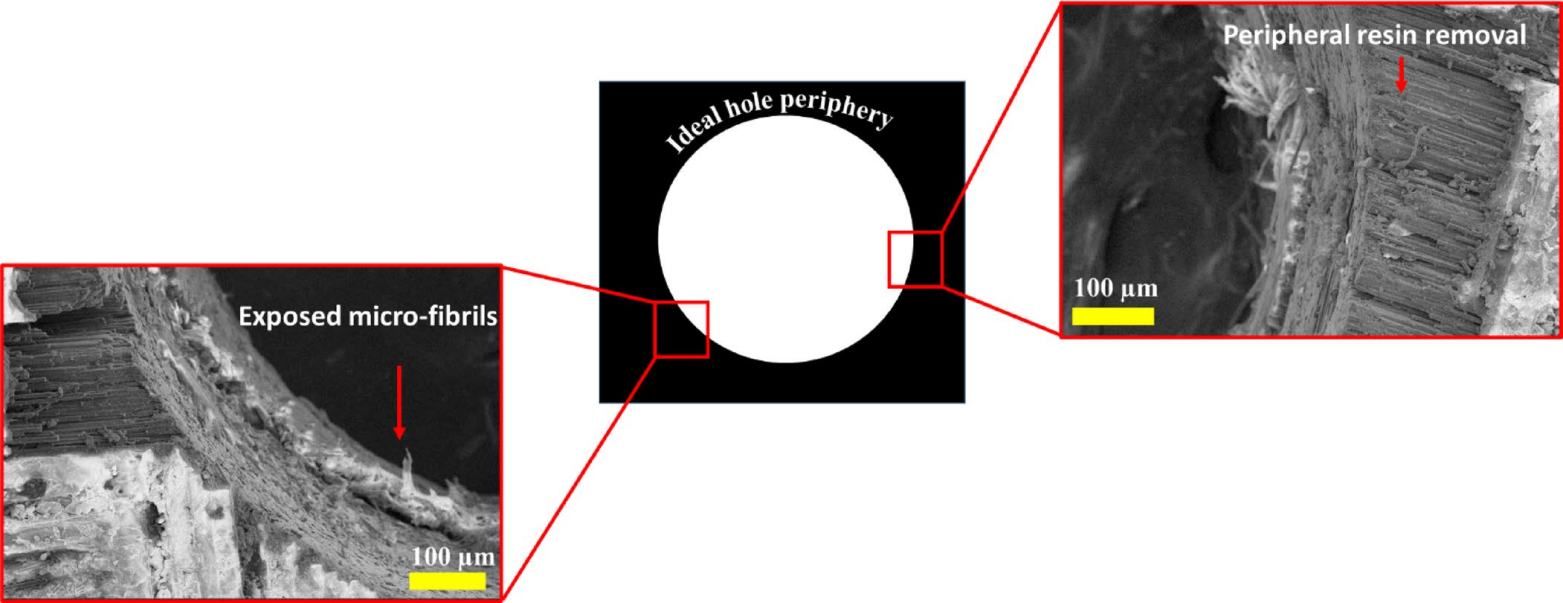
Micrographs showing exposed micro-fibrils and resin removal around the hole periphery.

The morphological assessment of the micro-holes was done using a scanning electron microscope (SEM). Figure 10 presents the surface morphology of the micro-hole at the entry. A visible amount of overcut is evident around the hole periphery, which can be correlated with the results obtained from optical micrographs, as discussed in the earlier section. The overcut during µEDM of CKHC can be attributed to the SS-sparking phenomenon, which is inevitable during the µEDM process^30^. A small amount of fiber protruding from the inside surface of the hole can be seen. These unremoved materials may be due to inefficient heat revied by the particular region of the workpiece. The CKHC is a combination of three different types of materials, viz. carbon fiber, Kevlar fiber, and epoxy resin. Carbon fiber demonstrates a relatively higher thermal conductivity (⁓7 W/mK)^31^ contingent upon fiber orientation and grade, hence promoting effective heat dissipation from the spark zone. This leads to localized yet expedited heat transmission, enhancing material removal efficiency. Conversely, Kevlar fibers exhibit markedly poorer thermal conductivity (0.04 W/mK)^32^, rendering them ineffective heat conductors. This results in localized thermal accumulation during discharge, frequently causing deeper craters and increased thermal damage due to inadequate heat dispersion. The epoxy matrix exhibits thermal conductivity values ranging from around 0.2 to 0.4 W/mK^31^. It serves as a thermal insulator and causes irregular heat distribution during µEDM. The limited thermal conductivity of epoxy and Kevlar results in uneven melting and reduced material loss rates, but carbon fiber reinforcing improves the thermal responsiveness of the composite. These differences collectively influence the material removal during µEDM of CKHC. The morphologies of the micro-hole around the periphery are presented in Fig. 11. Exposed micro-fibrils are visible, which are the result of incomplete material removal. As the heat propagation in the CKHC is anisotropic in nature, some part of the workpiece receives more heat, which leads to excess material removal. This can be confirmed in Fig. 11, where unwanted removal of resin around the hole periphery can be observed.

## Conclusion

The micro-hole fabrication on CKHC was carried out in this experimental endeavor using µEDM. Through-holes could be successfully created with the aid of a rotating copper tool and conductive sheet to help with the sparking. The significant observations from the study are:

1. The Taguchi analysis revealed that the sets of the optimum values of input parameters for the minimum MT and maximum DOC are - voltage: (180, 120) volts, EDM feed: (2, 2) µm/sec, and tool speed: (1000, 1000) rpm.
2. The statistical significance of the regression model for MT and DOC was established, as evidenced by the high R-sq values for MT (0.8626) and DOC (0.9554). Voltage proved to have the highest impact on MT variation, while TS has the highest influence on DOC.
3. The ANOVA revealed that voltage has a contribution of 65.56% on MT, and TS has a contribution of 82.90% on DOC. EF was the least contributing factor for both MT and DOC.
4. The error values for MT and DOC, at optimum levels of input factors, as per the regression models were 3.96 and 0.93, depicting the goodness of the regression models.
5. The morphological assessment confirmed the dimensional deviation and consequent hole tapering due to SS-sparking. Phenomena like hole overcut, peripheral resin removal, and exposed microfibrils were visible in the SEM images.
6. The optimal setting of input parameters obtained from TOPSIS is V2/EF2/TS3, i.e. a voltage of 150 volts, EDM feed of 4 μm/sec, and tool speed of 1000 rpm. The confirmatory test showed an improvement of 0.757881 in the preferred solution for optimal settings as compared to initial settings.

This work successfully examined the µEDM machinability of CKHC, emphasizing the difficulties arising from the varying thermal and electrical characteristics of its constituent fibers. By utilizing single and multi-response optimization methods (Taguchi and TOPSIS) the study revealed the optimal process parameters that improve machining performance and surface quality. The assessment of the degree of circularity provided a novel perspective to the micro-feature fabrication in hybrid composites, yielding significant insights for microfabrication applications. Future work may be undertaken to examine the micro-milling characteristics of CKHC using varying tool materials along with various machine learning optimizations. While MT and DOC were designated as major metrics for assessing micro-feature integrity in the current study, it is advisable to use MRR in future research to more accurately measure process productivity and overall efficiency.

## Data Availability

The datasets used and/or analysed during the current study available from the corresponding author on reasonable request.
